# Awareness, Diagnosis and Management of Idiopathic Intracranial Hypertension

**DOI:** 10.3390/life11070718

**Published:** 2021-07-20

**Authors:** Rigmor Højland Jensen, Vlasta Vukovic-Cvetkovic, Johanne Juhl Korsbaek, Marianne Wegener, Steffen Hamann, Dagmar Beier

**Affiliations:** 1 Danish Headache Center, Department of Neurology, Rigshospitalet, University of Copenhagen, 1165 København, Denmark; vlasta.vukovic.cvetkovic.01@regionh.dk (V.V.-C.); johanne.juhl.korsbaek@regionh.dk (J.J.K.); 2 Department of Ophthalmology, Rigshospitalet, University of Copenhagen, 1165 København, Denmark; marianne.wegener.01@regionh.dk (M.W.); steffen.ellitsgaard.hamann@regionh.dk (S.H.); 3 Department of Neurology, Odense University Hospital, 5000 Odense, Denmark; Dagmar.beier@rsyd.dk

**Keywords:** idiopathic intracranial hypertension, awareness, management, organization

## Abstract

The diagnosis and management of idiopathic intracranial hypertension (IIH) can be difficult and multiple medical subspecialities are often involved. Several national and international guidelines regarding the investigations and management of IIH have been published in recent years but still there is no consensus about the optimal organization of IIH-care. The objective of this review was to propose and describe a referral pathway and an organization scheme for diagnosis and management of IIH. An extensive search of existing literature was conducted and summarized. In total, 237 IIH-articles were identified and hereof 43 included. The clinical practice in our specialized IIH-clinic is characterized and described. We conclude that an educational campaign involving medical care providers and patients with chronic headaches is necessary. A detailed organizational proposal for a referral pathway and management of IIH patients based on the literature search and our clinical experience from a highly specialized IIH outpatient clinic is suggested and discussed.

## 1. Introduction

The identification and management of idiopathic intracranial hypertension (IIH) can be challenging and various medical specialties are often involved. A clear referral pathway from the initial symptoms to the final, correct diagnosis and treatment is not well characterized. Several national and international guidelines regarding the investigations and management of IIH have been published in recent years [1,2] but there is still no consensus about the optimal organization of IIH-care [3]. There is also a paucity of studies in the literature on methods of monitoring disease progression in IIH [4].

With the close relation between IIH and obesity and the rapid increase in IIH incidence in parallel with pandemic obesity, the individual and the societal burden of IIH are also rapidly expanding as documented by Friesner et al. 2013 [5] and Mollan et al. in 2018 [6] and call for a concerted action.

Although the burden of IIH is alarming for the specialists within the field, we have to acknowledge that IIH remains a rare and often unknown disease in general practice and probably also occurs infrequently in most headache and eye clinics. A better quality of epidemiological studies is required to improve the burden of IIH and inform health policy about IIH management [7].

So, what makes a person with an undiagnosed IIH aware that something is wrong and urge them to consult medical help? Additionally, vice versa, what makes a healthcare provider aware that their patient might have IIH?

In addition, there is no early and reliable diagnostic test and the IIH-diagnosis is most often a diagnosis of exclusion. The revised diagnostic criteria from Friedman et al. [8] require papilledema and increased opening pressure based on a lumbar puncture. While this combination is very sensitive and specific in a specialized clinic, these criteria are difficult to apply to the vague symptoms that urge IIH-patients to their first medical consultations.

So, although early diagnosis and treatment is crucial for favorable visual outcomes there is also a risk for over- and misdiagnosis in as many as 40% of patients initially diagnosed with IIH, as documented by Fisayo et al. [9]. The potential consequences are substantial as years of unnecessary stigma, medical and/or even surgical treatment can be the result.

At this moment, there is very limited evidence for the exact referral pathways and in clinical practice patients often have a long history of consultations with multiple contacts before they receive a correct diagnosis and treatment, which can be crucial for their visual outcome. Therefore, more awareness about the disease is needed.

On this background, by the means of a literature search and clinical experience from our specialized IIH-clinic, we here aim to describe an organizational scheme for improved awareness, diagnosis and management of IIH.

## 2. Literature Search

We searched MEDLINE and Embase databases for articles on the management of IIH. The first database search was performed twice on 18 and 22 April 2021, with the following MeSH search string and no restrictions in terms of the date of publication: idiopathic intracranial hypertension AND management; idiopathic intracranial hypertension AND treatment; idiopathic intracranial hypertension AND organization. The database searches were limited to articles that were published as full text in the English language except for one in Spanish with an abstract in English, and included clinical trials, meta-analysis, randomized controlled trials, reviews and systematic reviews.

**Figure 1:**
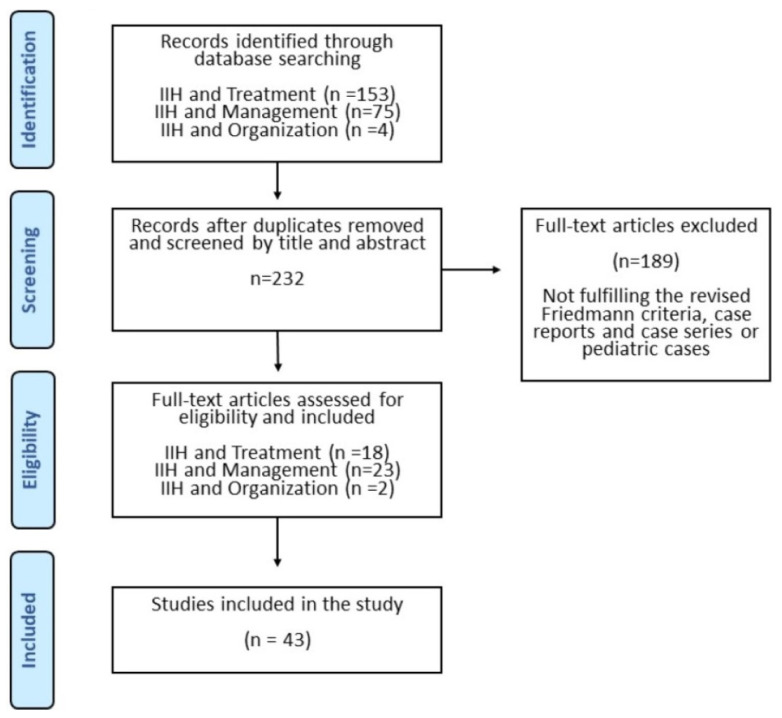
Results of the literature search.

In total, we reached 237 articles. Only studies on adults and studies using the revised Friedman criteria (2013) were included and we excluded case reports and case series. Finally, 43 were suitable for the purpose of the study (Figure 1).

In our literature search, we were unable to identify reports, surveys or national IIH campaigns targeting both the general public and/or health-care providers.

**Methods for “the clinical IIH setting”:**

Demographic data (sex and age) were retrieved from our clinical database (EPIC) system on patients referred to The Danish Headache Center under the diagnosis DG93.2 Benign Intracranial Hypertension.

## 3. Results

### 3.1. Literature Search

Forty three articles were identified and hereof 18 were focused on treatment, 23 on management and only 2 were related to organization and management. None were focused on awareness campaigns. Related articles from other specialties were thus included (Figure 1).

### 3.2. The Clinical IIH-Setting

The Danish Headache Center (DHC) is a public national referral center for severe and rare headache disorders and an integrated part of the Neurological Department, National Hospital, University of Copenhagen. The catchment area covers 2.7 mio inhabitants but patients can be referred from the entire country (total population in Denmark 5.8 mio). DHC has a highly specialized clinical IIH-function and offers multidisciplinary therapy within a team of headache nurses, psychologists and physiotherapist, if needed. Furthermore, DHC is part of a dedicated IIH team within the National Hospital in collaboration with three neuro-ophthalmologists, one dietician, two neurosurgeons and one orbital surgeon. An IIH-research group is closely connected to the IIH team.

**Figure 2:**
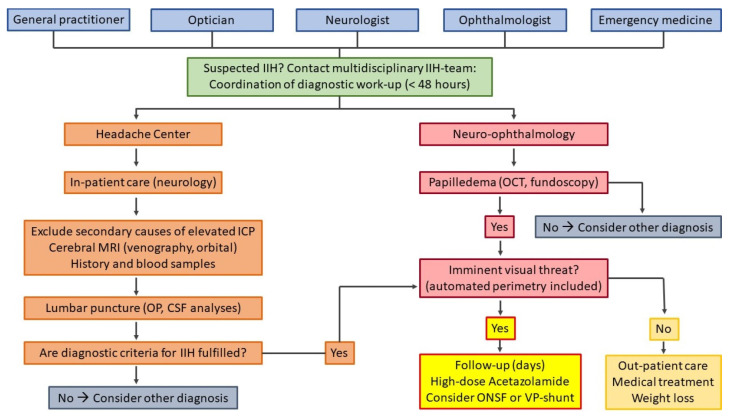
Clinical referral pathway for patients with suspected IIH.

The pathway for patients suspected of IIH is the following: general practitioners (GPs), neurologists, ophthalmologists, optometrists or other hospital departments refer the patients to the emergency room, the neuro-ophthalmology clinic or DHC. Hereafter, patients are admitted to our neurological department (Figure 2).

A detailed and standardized work-up is performed and if IIH is confirmed, an individualized treatment is planned within the IIH-team. Surgeons are informed the same day if there is an imminent vision threat or within 2 days if there is a significant progression despite medical treatment. Optic nerve fenestration (ONSF) is offered as the first line surgical treatment. In cases where ONSF does not provide improvement, the patient is referred to the second line surgical treatment, i.e., ventriculoperitoneal shunting. All IIH-patients are offered a concerted follow up program in DHC and the eye clinic, with weekly–monthly follow up visits in the beginning of the disease course depending on the severity of the disease and up to 6 month intervals in later long-term follow-up. During the follow-up period, supplementary fundus photography and visual field assessment are available in DHC using an automated fundus perimetry device (COMPASS), which includes laser ophthalmoscopy and in the case of doubt the output is discussed with a neuro-ophthalmologist, who will see the patient acutely, if needed.

Based on our recommendations that all suspected IIH patients should be referred to the DHC, the annual referral rate has doubled from 2017 to 2020. A total of 264 patients, 251 females and 13 males with an average age of 36.6 years suspected for IIH were admitted to DHC from January 2017 to March 2021. Hereof, only 133 patients (50.5%) fulfilled the criteria for IIH, whereas a variety of differential diagnoses were identified in the remaining patient group. This reflects the need for an IIH team that can provide an accurate diagnostic work-up and confirm or exclude the IIH diagnosis without delay. In the same period of time, a total of 10 IIH-patients (5.1%) were admitted to surgery: eight to ONSF and two to VP-shunts [10].

## 4. Creating an Optimal Diagnostic Process for IIH

### 4.1. Awareness and Education

The main barrier for an early detection and diagnosis of IIH is probably a lack of general knowledge of the disease. In our literature search, we were unable to identify reports, surveys or national IIH campaigns targeting the general public and/or health-care providers and to the best of our knowledge there are no existing campaign models. On the contrary, other awareness campaigns have been successfully conducted in the headache field with the initiatives of “Lifting The Burden: The Global Campaign against Headache” [11,12], which aimed to reduce the burden of headaches worldwide. In describing and evaluating other awareness campaigns, elements of a model developed by the Center for eHealth and Wellbeing Research could eventually be applied here [13]. This model consisted of five components: contextual inquiry, value specification, design, operationalization and evaluation and has been successfully used in other disorders such as diabetes [14] and medication overuse headaches [15].

The first step in an IIH awareness campaign should target the primary and secondary care level, the GPs, ophthalmologists, the optometrists, neurologists and the ear–nose–throat (ENT) specialists. There is no need to address the general population as the disease is still relatively rare compared to other more prevalent disorders, such as migraines and medication overuse headaches [15].

Further, if the health care providers do not know the symptoms or the disease there will be no link to a diagnostic process. With the model described above a survey of the general knowledge of the disease, followed by written and spoken educational information (leaflets, videos, webinars, guidelines and scientific publications) and followed by a new evaluation survey could be a useful model to pursue.

However, such educational and awareness campaigns should be implemented in the curriculum for medical education and repeated on a regular basis to gain enough impact. In an interesting approach from the Birmingham IIH group, patients and physicians were also engaged to delineate their research priorities and their most important priorities were to understand the etiology and management of IIH [16]. The reflections and inspiration from such experienced IIH-patients could benefit an educational campaign aimed for health care professionals and in a later stage maybe also benefit a concerted patient and more public oriented campaign. For a summary of the target points of an IIH awareness program, see Table 1.

The majority of IIH patients report that they must consult a high number of health care professionals before the right diagnosis is established. That is probably due to the lack of education as mentioned above but also due to the broad variety of symptoms they are presented with in the early stage. Here more education and more awareness of IIH among health care providers could be beneficial for the recognition of IIH but many GPs, private practicing ophthalmologists and neurologists will probably still be unable to manage these patients in their settings. Therefore, referral to a headache center with a specialized IIH team is recommended.

### 4.2. Referral Pattern

The IIH patients that present a headache phenotype that mimics a chronic migraine and/or chronic tension-type headache may be exposed to migraine preventive medications and triptans for months or even years, but not treated for the underlying disease, and thus are at risk of permanent visual damage.

For this reason, there is an imminent need for a clear referral pathway to a specialized care setting. Thus, when a clinician suspects IIH, the patients should be referred to a specialized multidisciplinary center for IIH. In this case it could be a headache center, an ophthalmological center or other settings with special interest in IIH. In the case of the presence of the two cardinal symptoms and signs, a headache and papilledema, the suspicion of intracranial hypertension, either secondary or primary, should always be raised. The diagnosis of IIH without papilledema (IIHWOP) is more challenging as the condition is rare and the neuroradiological signs are open for individual interpretation [17]. Thus, the diagnostic process in IIH requires careful insights in the multitude of differential diagnoses and a careful work-up is essential to avoid the significant risk of both an over- and underdiagnosis [9]. As shown above, half of the referred patients to the DHC did not fulfill the IIH diagnosis and might have been misdiagnosed in another setting. In particular, atypical IIH patients, i.e., males, are in danger of presenting IIH in an advanced stage of the disease. Males with IIH are twice as likely as females to develop severe visual loss and males often have atypical symptom profiles.

### 4.3. Early Diagnosis

The main targets in the early phase of the disease are to identify the disorder and preserve visual function.

If a headache is the main symptom, the pathway from the GP to the neurologist and thereafter to a headache specialist can be long and troublesome. The early symptoms can be unspecific and vague and IIH in the early stages can be difficult to recognize. The cardinal symptom, a headache, affects up to 90% of IIH patients [18], but is also highly prevalent in the general population. In the International Headache Classification 3 edition (ICHD3 criteria) [19] regarding headaches attributed to IIH it is stated in criteria C as follows the “headache has developed or significantly worsened in temporal relation to the IIH, or led to its discovery”. In practice, the precise start or the temporal relationship can be very difficult or even impossible to establish, as the disease does not have an abrupt start and may have developed gradually over weeks and months. To be helpful in the clinic, ICHD3 criteria need validations as was the case with the previous diagnostic criteria [20,21,22].

Migraines affect up to 16% of the general population and tension-type headaches up to 76% [23,24], and both primary headache disorders affect primarily the same population group as IIH, namely young females. As migraines also are associated with obesity, especially the chronification hereof [25,26], the headache attributed to IIH may thus mimic a chronic migraine. The main difference between a migraine and IIH related headache is that in most IIH patients, a “new” headache has developed over a relatively short time, in many but not in all cases being orthostatic or worse in recumbent position. The headache is mostly described as tension-type-like, holocephalic and continuous. It is typically increasing over weeks to include more migraine-like features, such as nausea and photo- and phonophobia but in most cases widely it is unspecific and may be difficult to classify.

The initial visual disturbances that most patients with IIH may be presented with are vague, unspecific and sporadic. Patients complain of blurred vision, but this is also reported in 47% of other headache patients [22]. Further, blurred vision may mimic a wide variety of other conditions including refractive anomalies or even a migraine aura. Later in the disease stage of IIH the visual problems become more specific and may include transient visual obscurations (TVO), double vision and decreased acuity but surprisingly a high number of our patients are still diagnosed in a very late stage with severely impaired visual fields.

The diagnosis of papilledema is not always easy, especially in the emergency room and/or in the headache clinic. Many young doctors are unfamiliar with direct ophthalmoscopy and may overlook a mild papilledema. On the other hand, many optic disc anomalies might erroneously be misdiagnosed as papilledema, even by ophthalmologists. The most common diagnostic error in the diagnosis of IIH is actually inaccurate ophthalmoscopic examination in headache patients [9]. On top of the problematic identification of papilledema, many differential diagnoses, such as tight discs, tilted discs, buried optic disc drusen or optic neuropathy with a swollen optic disc due to another pathology other than intracranial hypertension, can mimic a papilledema. Although suggestions for a practical approach to these patients have been published, there is a significant need for trained neuro-ophthalmologists to confirm the diagnosis [3].

Optical coherence tomography (OCT) is a rapid, non-invasive imaging technique increasingly used among private ophthalmologists and it is the standard in all eye departments. Although several pitfalls exist, OCT is probably one of the most useful tools to identify and quantify papilledema in IIH and provides important clues, such as peripapillary retinal nerve fiber layer (RNFL) thickness increase, peripapillary choroidal and/or retinal folds and wrinkles, which all point in the direction of true papilledema [27,28].

The diagnostic value of the optic nerve sheath diameter using an ultrasound could also be a valuable and atraumatic diagnostic tool for IIH [29,30,31] but further validation studies are needed.

In recent years a major technological development in cameras have enabled opticians to make high quality fundus photos and thereby identify suspected papilledema. A more widespread use of fundus photos in headache clinics and emergency rooms may also contribute to early screening and identification of suspected papilledema, especially if there is lack of access and/or long waiting time for neuro-ophthalmologists and headache specialists.

### 4.4. Other Symptoms

IIH-patients may also report a variety of other unspecific symptoms, namely back and neck pain, dizziness, pulsatile tinnitus and sometimes cognitive symptoms like memory impairment and concentration difficulties [32,33,34]. The physician should not expect the patient to name all symptoms but must ask about the presence of each one.

### 4.5. Neuroimaging

Neuroimaging is required for the exclusion of secondary causes of intracranial hypertension. Neuroradiological evaluation of signs suggesting elevated ICP can be complex and needs a specialized neuroradiological evaluation. The present neuroradiological criteria are only applied for IIH WOP and not for the more classic IIH patients with papilledema [8]. Thus, there is a high risk of misdiagnosis in IIH so a very careful evaluation and validation of the present methodologies could be of important value for the diagnostic process. The topic is well described and discussed by neuroradiology experts elsewhere.

### 4.6. Lumbar Puncture

Measurement of opening pressures and CSF analysis is required for a precise diagnosis but a lumbar puncture can be a challenging procedure. Patients often are afraid of lumbar punctures due to the myths about pain, repeated unsuccessful attempts and the risk of post lumbar puncture headaches [31]. Likewise, some doctors may be reluctant to perform lumbar punctures because of a lack of skills and practice, especially in very obese patients. The use of ultrasound guidance and atraumatic needles could benefit the patients due to more precise access and minimize the risk of a post lumbar puncture headache [32,33].

Furthermore, the measurement of opening pressure presents another common reason for overdiagnosis, as it is a snapshot and not always a true reflection of the real intracranial pressure [34,35]. Positioning of the patient in the lateral decubitus position with stretched legs and neck is important to avoid falsely elevated readings [34].

Thus, there is a huge unmet need for better, valid and atraumatic diagnostic tools for IIH, especially in the early disease stages but certainly also later for follow-up visits.

### 4.7. Additional Blood Tests

In a minority of suspected IIH patients, an atypical presentation can be seen. These are mostly young/elderly females with normal weight or males. In these cases, it is important to exclude secondary causes, such as rheumatological or infectious diseases. Additional blood tests are required and, if positive, referral to specialists is needed.

The target points for the diagnostic process are summarized in Table 2.

## 5. Management and Follow-Up

When the patient has received the correct diagnosis, the question about treatment is naturally asked. The main target is to preserve vision but also to reduce the headache and the other debilitating symptoms. The present recommended strategies are a combination of medication, life-style changes and weight loss in the vast majority of patients. Suggestions for management have been published [1,2] but only a few randomized, controlled trials are currently available. The Cochrane review from 2015 was therefore inconclusive with no clear recommendations [35]. Thus, most existing treatment strategies are based on clinical practice and local consensus but not on large-scale treatment trials [36].

### 5.1. Medical Treatment

Effective treatment is an unmet clinical need in IIH. Current medical management remains centered around weight management, which is challenging. Identification of novel molecular targets thought to underlie IIH pathology is now being translated to clinical trials [37,38].

The most frequent medication used in treating IIH, acetazolamide, is unspecific and is not well tolerated, causing up to half of the patients to discontinue their treatment [39,40]. Topiramate has been tested in an animal study and seemed more efficient than acetazolamide to reduce ICP but so far no randomized controlled human studies are available [41]. For many patients topiramate reduces appetite—a side effect than can be helpful in weight reduction. However, adherence to topiramate are likewise hampered by significant side effects as paresthesia, nausea, sedation, taste alteration and kidney stones and cognitive impairment [39,40]. Careful education in symptoms and signs is therefore of immense value for these patients as adherence to medical treatment can be determinant for the beneficial outcome.

### 5.2. Obesity and Other Comorbidities

Comorbidities to obesity are well documented, such as depression and anxiety [42]. However, medication used to treat these disorders can also have undesired weight gain and metabolic syndromes as side effects.

Since obesity and recent weight gain are major risk factors, weight management is always recommended and an important part of the management of all patients [42,43,44]. Psychiatric disorders itself often make it impossible for the patients to succeed in weight loss and especially if there are concurrent eating disorders, so the interdisciplinary can also be beneficial here.

Obesity is in itself a disorder that has a complex pathogenesis and can be caused by genetic disposition or psychiatric disorders but the rapid increase of obesity can probably best be explained by environmental factors in terms of easy access to food rich in fat and sugar combined with lifestyle changes towards more immobility. Reducing weight may thus seem the most obvious goal to set but maintenance of weight loss is the main challenge both in IIH and in obesity in general [44].

### 5.3. Surgical Management

In the subset of patients presenting with an imminent visual threat it is urgent to identify and refer them to the right surgical intervention, either shunts, optic nerve fenestration or venous stents.

The surgical procedures are needed when vision loss is rapidly progressing and medical treatment and weight loss has proven insufficient. The advantages and disadvantages have been extensively studied and also reported in this issue [10]. However, here it is also extremely important with a concerted multidisciplinary IIH-team in a specialized setting as we presented. We found it to be crucial to have a very careful and frequent even daily or weekly ophthalmological follow up in order to identify these malignant cases. Apart from a few studies on high baseline concentrations of the neurofilament light chain, indicating a risk of optic nerve atrophy and high BMI as an indicator for poor visual outcome, respectively, we still do not have clear prognostic predictors at the time of diagnosis [45,46,47,48].

An early surgical intervention should definitely be avoided in the majority of cases, especially if the IIH-diagnosis is unconfirmed, the vision is not acutely threatened and a possible secondary cause as cerebral venous thrombosis or other organ diseases is not investigated or identified. Further, the effect of surgical interventions on the headache outcome has not been confirmed and therefore the general recommendation is that surgical interventions should be reserved for patients with an imminent threat to their vision and are not needed for the headache [49]. In addition, it is the authors personal experience that the patient–doctor alliance can be difficult to obtain or maintain after surgical procedures, especially shunting procedures. Although unsubstantiated by data the patient focus is more directed to an eventual mechanical shunt dysfunction than to more conservative treatment strategies as life-style changes, weight loss or medication. In a study from Philadelphia [50] it was documented that the total use of surgical treatment of pseudotumor cerebri numerically were increased but not relatively (especially the use of VP-shunts compared to ONSF) in relation to the overall very high number of hospitalizations. Thus, they concluded that these trends reflected the improvement in their set-up, the medical treatment practice and outcome. If the relative number of surgical interventions can be minimized and directed to the right patients with an imminent risk of vision loss and not to those that could be managed medically, hopefully the overall disease burden can be reduced by better organization and medical treatment. This is clearly in line with our clinical practice where only eight ONSFs and two VP-shunts have been performed in the past 4 years [10]. A detailed case to case discussion and a direct contact to a dedicated surgical IIH-team have proved to be very useful in our setting.

### 5.4. Follow Up

From the above it is obvious that uncertainty about the cause(s) in IIH exists and that the evidence for treatment is evolving but not yet specific or firm. For the patient getting the correct diagnosis and a treatment plan this can also be a challenge. It is therefore essential with long term follow up until the situation is stable and patients are properly educated in their condition.

Based on the estimation of the stage and burden of the disease, an IIH patient can be followed-up more closely in the beginning (3–5 days) and in later stages with more remote controls. In cases of visual improvement on the first two controls, which are 2 months apart in most cases, further controls can be with 3–4 months intervals. High CSF opening pressure, worsening vision/papilledema, rapid progression, severe papilledema with greater RNFL thickness and abnormal VEPs may be some of the alarming signs for physicians, but none of these parameters can be used as an independent predictor for visual outcome in isolation. Visual field loss at presentation is probably the most important predictor of the final visual outcome in these patients [51,52]. The target is to provide the best treatment and assure that the vision is not endangered.

Furthermore, relief from headache and additional symptoms is warranted. Close monitoring of laboratory parameters is needed (potassium, eGFR and hydrogen-carbonate), especially if acetazolamide is introduced at a high dosage. Potassium substitution and in some cases sodium bicarbonate are needed, too. During the follow-up, especially in the first phase, the IIH specialist, the IIH nurse and the ophthalmologist should have a possibility for close consultations and adjustments in the treatment plan.

## 6. The Importance of Specialized Teams and Multimodal Management

As the disorder is so complex and can be difficult to manage, a multimodal treatment strategy is needed and a dedicated team consisting of neurologists, neuro-ophthalmologists, neurosurgeons and dieticians are therefore recommended in most guidelines. The documentation for these multimodal structures is now evolving. By means of a structured follow-up protocol at 3, 6 and 12 months for IIH in the neuro-ophthalmological unit in a tertiary hospital in Spain the number of LPs was significantly reduced, the outcome was markedly improved. Thus, it was concluded that optimization of follow-up could lead to reduced use of resources and better care [53]. Likewise, in the long-term study the visual prognosis was very good after a regular and structured follow-up program in our dedicated neuro-ophthalmological team setting [51].

It is also important to address the patient’s comorbidities such as depression or eating disorders, along with help from the dietician but more evidence is also needed here.

The management of an IIH-headache is often reported to be complicated and for unknown reasons the headache does not follow the visual improvement. When the intracranial pressure is normalized, usually the diplopia, tinnitus and neck pain resolve, whereas the headache does not improve to a similar level [48,52,53]. In the study by Yri et al. a similar outcome was reported and as many as 50% of IIH patients still suffered from a daily or near daily headache after treatment whereas the visual prognosis was very good to excellent [54]. Moreover, a significant number probably continues to suffer from cognitive deficits that may complicate adherence to treatment and/or to their former work [55,56].

Thus, there is an unmet need for a mechanistic insight and larger randomized treatment studies with a focus on the entire IIH-disease as the present strategy on the visual outcome and headache phenotype alone may be incomplete. The target points of multimodal management are displayed in Table 3.

## 7. Conclusions and Perspectives

Much more awareness about IIH is needed as the disease still is relatively unknown, at least in the primary and secondary health care system and in the general population. It is well documented that an early diagnosis leads to a better visual outcome but there is a significant risk of misdiagnosis and under- and overtreatment. On the basis of the literature and our experience, a structured educational campaign and a clear referral pathway to highly specialized national IIH-centers are suggested. Such initiatives may ensure better care and outcome but proper large-scale testing hereof is needed. Further, it is important to have a certain critical mass of IIH patients. A clear referral pathway to a specialized center for suspected IIH will enable the physicians to revisit the multiple differential diagnoses that affect up to half of our patients. Further a specialized IIH center can and should also be a promotor for research and the development of new and better treatment strategies.

## Figures and Tables

**Table 1:** Target points of the IIH awareness program.

Target Points of the IIH Awareness Program
- Address GPs, general neurologists, ophthalmologists, optometrists and the ear–nose–throat (ENT) specialists, followed by written and spoken educational information (leaflets, videos, webinars, guidelines and scientific publications)
- Re-evaluation after 6–12 months
- Additional education based on re-evaluation results
- Address the patients with chronic headache—awareness of a possible other diagnosis than chronic migraines or tension type headaches
- Within home-websites (headache clinic and hospital) address a special section for IIH

**Table 2:** Target points of the diagnostic process.

Targets Points of the Diagnostic Process
- Improve history taking (prepared questionnaires sent to the patients before they meet for the first consultation and prepared questionnaires for doctors who are taking the history), especially in patients with chronic headaches
- Education of doctors, especially in an emergency room setting in direct ophthalmoscopy
- If possible, provide access to a fundus camera or a scanning laser ophthalmoscope
- Education of doctors in the accurate LP procedure
- Establish cooperation with a neuroradiologist specialized in IIH

**Table 3:** Target points of multimodal management.

Targets Points of Multimodal Management
- Establish an IIH team in the headache center or regional hospital (headache and nurse specialists for IIH)
- Establish cooperation with a nurse trained in IIH problems whom the patients can reach on a daily basis
- Establish reliable cooperation with ophthalmologists, if possible, neuro-ophthalmologists
- Establish reliable cooperation with neurosurgeons educated in IIH
- Provide access to neuro-ophthalmologists and neurosurgeons for acute referral, also during weekend and holidays
- Provide access to dietician
- Provide access to physiotherapists (aim is guidance to applied physical activity for patients)
- Establish cooperation with psychologists and/or psychiatrists for the treatment of comorbidities
